# Adolescent and young adult (AYA) patient involvement and engagement in European health care and research projects: expanding the scope of patient advocacy

**DOI:** 10.1016/j.esmoop.2025.104478

**Published:** 2025-02-24

**Authors:** U. Košir, F. Lysen, N. Unterecker, T. Deželak, E. Sturesson, I. Shakhnenko, D. Stark, K. Rizvi, A.-S. Darlington, L. Wee, W.T.A. van der Graaf, O. Husson

**Affiliations:** 1 Youth Cancer Europe, Cluj-Napoca, Romania; 2 Department of Society Studies, Maastricht University, Maastricht, The Netherlands; 3 International Affairs & Policies, European Organisation for Research and Treatment of Cancer, Brussels, Belgium; 4 Teenage and Young Adult Cancer Research, Leeds Institute of Medical Research, Leeds, UK; 5 School of Health Sciences, University of Southampton, Southampton, UK; 6 Faculty of Health, Medicine and Life Sciences, Clinical Data Science, Maastricht University, Maastricht, The Netherlands; 7 Department of Medical Oncology, Netherlands Cancer Institute, Antoni van Leeuwenhoek, Amsterdam, The Netherlands; 8 Department of Medical Oncology, Erasmus MC Cancer Institute, Erasmus University Medical Center, Rotterdam, The Netherlands

**Keywords:** advocacy, patient involvement, health literacy, adolescent and young adult, AYA, cancer

## Abstract

Patient involvement and engagement (PI&E) in health care and research has gained prominence, shifting towards person-centred approaches and shared decision making. Patients actively participating in health care design and research lead to better quality and efficiency of care. However, implementing meaningful PI&E is challenging and requires adequate resources and evaluation frameworks so that it does not result in tokenism. This is particularly important when considering niche areas like adolescents and young adults (AYAs) with cancer. As AYAs’ unique needs continue to gain recognition, it is becoming increasingly important to incorporate their expertise and diverse perspectives in navigating care. Large-scale European consortia that focus specifically on AYAs offer opportunities to establish successful partnerships with AYAs in the design and creation of the next generation of equitable, diverse, and inclusive cancer care. Concrete actions for meaningful AYA PI&E are discussed.

## Highlights

• Effective AYA involvement and engagement enhances health care quality and efficiency.
• Research consortia enable AYAs’ meaningful integration in all research and development phases, recognising their expertise.
• Cancer diagnosis is just one aspect of young people’s identity.

Patient involvement and engagement (PI&E) has become increasingly common in health care organisation, delivery, and research.^1^, ^2^, ^3^ In traditionally disease-centred and top-down health systems, the paradigm is shifting towards person-centred approaches and shared decision making.^4^ In this transition, involving the end-users, namely patients, is considered a key to success. When health care services, from primary prevention to palliative care, are designed with careful consideration of patient needs and preferences, the outcomes have been seen to be of better quality and to be more efficient and sustainable.^5^^,^^6^ Including patients and their caretakers in designing, governing, and practicing health care can also expand patients’ ability to choose, democratise decision making, emancipate patients, and advance equitable and just medicine.^7^, ^8^, ^9^, ^10^ Moreover, engaging patients in health research can result in improved recruitment and retention of participants, enhanced study design, and research that aligns more closely with stakeholders’ needs.^11^

Although it might be easy to subscribe to the idea of PI&E in theory, it is far more difficult to implement in practice.^12^ The barriers to a meaningful PI&E are many and multifaceted, not least the absence of consensus around the term.^13^ Bringing PI&E into the existing health care systems requires time, adequate resources, including sufficient funding,^11^ and additional training of health care professionals,^14^ all of which require attention and may compete with limited capacity and other priorities within a given health care setting. Moreover, there is a lack of evaluation of the costs and justifications of patient involvement in health care, as well as a lack of consensus on how to define and evaluate the efficacy and quality of engagement practices.^15^^,^^16^ At present, the majority of PI&E practices still primarily translate into gathering feedback or unidirectional information provision, while in planning research and study protocols the norm remains consultation rather than co-design across all stages of work.^12^^,^^17^^,^^18^ Poor participatory approaches may even have harmful effects, such as ‘tokenism’,^12^^,^^19^ when engagement legitimises innovation without the ‘meaningful engagement’ with patient experiences and needs that would provide the greatest benefits.^20^ Particularly in medical research, when we still frequently speak of research subjects rather than research participants or partners, it is easy to fall victim to tokenising a few willing individuals and have them fulfil a requirement (i.e. tick a box) instead of genuinely empowering them to truly engage in the research process, enabling them to contribute meaningfully.

The work of patient advocacy groups and non-governmental organisations is invaluable in realising meaningful PI&E practices. These organisations can act as monitors and promoters of PI&E in all stages of research and care and place focus on empowering individuals to contribute even when they are isolated due to their background, underrepresented status, or illness. Increasingly, PI&E has become institutionalised in health care and research practices. Various formats for participation have become professionalised and integrated into research and grant-application infrastructures, for example, in hospital budgeting decisions, drug development, health technology assessment, medical education, and evaluation of health care services.^21^, ^22^, ^23^ Some institutions have not only adopted policies that endorse PI&E [e.g. North American Primary Care Research Group (NAPCRG), https://napcrg.org/home/] but even formally require patient engagement [Patient-Centered Outcomes Research Institute (PCORI)].^10^

Having adequate representation of a group of patients can be particularly challenging when studying or working in a niche or rare disease area. Adolescents and young adults (AYAs) with cancer have now been recognised as a unique group of patients, often navigating the poorly defined zone between paediatric and adult services. Because cancer was traditionally considered a disease of older age, AYAs’ unique developmental needs and challenges such as compromised fertility, sexuality or family planning, and educational or vocational aspirations were often brushed aside resulting in many unmet needs.^24^ Over the past years, there has been a growing focus on balancing survival as the primary clinical endpoint with the often compromised psychosocial well-being, which occurs in one-third of AYAs.^25^ Despite increased survival, young people living beyond acute cancer treatment often report poor mental health, lower quality of life, financial discrimination, difficulties in returning to professional paths, compromised fertility, long-term treatment effects, and even lifelong disability.^24^^,^^25^ However, despite the increased recognition of AYAs, new findings or improvements are often limited to Western, developed, or affluent settings. Although engaging AYAs into clinical trials is becoming more common,^26^ conducting novel research can also be difficult due to fragmented or non-specific patient-reported outcome or experience measures.^27^^,^^28^ As a result, many academic publications and international working groups of experts expressed support and outlined the need for strong partnership with AYA patient advocates,^24^ but the question remains: How do we get AYAs to participate meaningfully in research and policy, instead of repeating what we already know is needed?

The key aspect that may overcome tokenism and begin ‘walking the talk’ is to consider young people as whole and acknowledge the experiences and expertise in navigating cancer when young as integral to and foundational within building AYA cancer health care and research.^29^ Perhaps we are moving forward; from identifying that AYAs’ needs are unique, to consulting them on projects, research, and clinical decisions. However how do we truly empower them? One aspect to the answer is simple—consider AYA as possessing equal expertise, across the range of decisions that are needed to deliver optimal cancer care. We can acknowledge that AYA cancer survivors’ years of experience navigating health care have made them experts, in their own care and needs. For as long as we limit ourselves to the diagnostic labels (‘a person living after bowel cancer’ or ‘a person living with kidney damage after cervical cancer’), we not only risk losing the engagement of many people who prefer to no longer identify with their previous cancer, but also we overlook the opportunity to move that illness into the background and instead bring forth a person with all their knowledge and expertise—be it medical or not—that will have been informed by their cancer experience. In the process of building improved AYA care infrastructures and communities that warrant AYA expertise and experience, an absolutely vital element is to actively empower and incorporate AYAs from minority groups. Marginalised AYA patients may face considerable additional barriers and burdens when engaging in patient involvement. Therefore, new initiatives should allocate resources to operationalise existing expertise in fostering equity, diversity, and inclusion (EDI) in PI&E, and in particular, they should materialise recent recommendations aimed at advancing greater inclusivity of racial and ethnic minority AYAs.^30^

Any healthy partnership takes time to develop and is a two-way street. The Europe’s Beating Cancer Plan and resultant research support and capacity provide invaluable opportunities for AYAs facing cancer. When youth organisations are put in the driving seat, we begin seeing positive changes. For example, European Union (EU)-funded EU-CAYAS-NET (https://beatcancer.eu/) is a resource platform for AYAs created by AYAs. The project’s mission has encouraged the regulators to require that subsequent financial support for the continuation of EU-CAYAS-NET is conditional on PI&E—the AYAs need to remain in the driving seat. Although this project is yet to endure the test of time, the fact that AYAs are co-creating novel methods such as peer visits (https://osf.io/apgm5), where AYAs act as peer researchers and observers, is likely to be a contributing factor to sustainability of such work. Another example is the STRONG-AYA (https://strongaya.eu/) international consortium, a project using novel technological and PI&E techniques to overcome current challenges such as the lack of standardised measures and data collection and sharing, across borders. In STRONG-AYA, patient-experts work as an integrated partner, through advisory boards. All of the key stakeholders and researchers turn to the advisory board to develop research questions and data ‘use cases’, promote research, design the web-based and professional platforms, and consult about acceptable practices in the realm of digital data. These are just a few examples of how successful partnerships can shape up.

Based on our learnings, we recommend the following become the norms:

1. Ensure that AYAs actively contribute to every stage of future research proposals, including drafting sections, rather than solely providing feedback. Hold funding-seekers responsible for maintaining a transparent record of this collaborative process.
2. Dedicate aspects of research or service redesign that are to be run by, not merely with, AYAs—they can be independent and dependable, willing to learn like all of us.
3. Leverage AYAs’ skills beyond their health care navigational abilities as their identities do not revolve around illness alone. Therefore, why hire external graphic designers, information technology support, nutritionists, and lawyers when they might be available through AYA participation, with their skills enhanced by the expertise of a patient’s perspective?
4. Offer payment and flexible working and compensation options, acknowledging that AYAs are sharing their expertise and as such, they deserve fair remuneration. Collaborate with them to determine the best compensation methods.
5. Allocate funding to facilitate the implementation of effective strategies for PI&E in European health care and research projects. Including patients in the development process will enhance EDI in the development of new AYA cancer care infrastructures.

Over time and with continuous support of such efforts that acknowledge patient expertise and allow us to improve upon traditional top-down approaches, diverse groups of AYAs can finally engage and become part of governing and designing care and research practices. When care is designed with cancer in the background and the person in the foreground, this will speak the language of larger communities, and make new developments in AYA cancer care self-sustainable, more approachable, and more suitable for long-term follow-up, where life continues. After all, the goal is living beyond, not just surviving the acute cancer treatment.

Some of the old paradigms need to be broken down, for new ones to rise. Although new approaches can be met with resistance, it is precisely AYAs’ resilience that can bring the next wave of positive change. With increasing complexity of the health care systems, designing health care with patient expertise will not only make it better for them, but also result in higher value health care, improved health literacy, and health system navigational skills, all of which ultimately contribute to a healthier society.

In academically oriented work, especially psychosocial research, we mostly want to measure, quantify, and observe partly intangible entities. Despite the best checklists or questionnaires, or even novel funding agency requirements (perhaps only resulting in an increased bureaucratic burden), we cannot always guarantee meaningful engagement with young people with lived experience of cancer. The real Engagement—with a capital E—will occur when shared decision making and meaningful dialogue will no longer be prescribed, but occur as the only natural way. This can only result from the recognition and acceptance of our shared humanity, and that it is almost by sheer chance that one of us is sitting on one side of the consultation desk and not the other. However, until PI&E becomes an inherent part of the trade, we must remain strategic and deliberate.

## References

[1] Bagley H.J., Short H., Harman N.L. (2016). A patient and public involvement (PPI) toolkit for meaningful and flexible involvement in clinical trials – a work in progress. Res Involv Engagem.

[2] Baines R.L., Regan De Bere S. (2018). Optimizing patient and public involvement (PPI): identifying its “essential” and “desirable” principles using a systematic review and modified Delphi methodology. Health Expect.

[3] Ocloo J., Garfield S., Franklin B.D., Dawson S. (2021). Exploring the theory, barriers and enablers for patient and public involvement across health, social care and patient safety: a systematic review of reviews. Health Res Policy Syst.

[4] Tonelli M.R., Sullivan M.D. (2019). Person-centred shared decision making. J Eval Clin Pract.

[5] Marzban S., Najafi M., Agolli A., Ashrafi E. (2022). Impact of patient engagement on healthcare quality: a scoping review. J Patient Exp.

[6] Bombard Y., Baker G.R., Orlando E. (2018). Engaging patients to improve quality of care: a systematic review. Implement Sci.

[7] Del Savio L., Prainsack B., Buyx A. (2016). Crowdsourcing the human gut. Is crowdsourcing also ‘citizen science’?. J Sci Commun.

[8] Prainsack B. (2017).

[9] Brett J., Staniszewska S., Mockford C. (2014). Mapping the impact of patient and public involvement on health and social care research: a systematic review. Health Expect.

[10] Coulter A., Ellins J. (2007). Effectiveness of strategies for informing, educating, and involving patients. BMJ.

[11] Brett J., Staniszewska S., Mockford C. (2014). A systematic review of the impact of patient and public involvement on service users, researchers and communities. Patient.

[12] Ocloo J., Matthews R. (2016). From tokenism to empowerment: progressing patient and public involvement in healthcare improvement. BMJ Qual Saf.

[13] Gibson A., Britten N., Lynch J. (2012). Theoretical directions for an emancipatory concept of patient and public involvement. Health.

[14] Herrin J., Harris K.G., Kenward K., Hines S., Joshi M.S., Frosch D.L. (2016). Patient and family engagement: a survey of US hospital practices. BMJ Qual Saf.

[15] Staley K. (2015). ‘Is it worth doing?’ Measuring the impact of patient and public involvement in research. Res Involv Engagem.

[16] Mockford C., Staniszewska S., Griffiths F., Herron-Marx S. (2012). The impact of patient and public involvement on UK NHS health care: a systematic review. Int J Qual Health Care.

[17] Wait S., Nolte E. (2006). Public involvement policies in health: exploring their conceptual basis. Health Econ Policy Law.

[18] Shakhnenko I., Husson O., Chuter D., Van Der Graaf W. (2024). Elements of successful patient involvement in clinical cancer trials: a review of the literature. ESMO Open.

[19] Evans J., Papoulias S.C. (2020). Between funder requirements and ‘jobbing scientists’: the evolution of patient and public involvement in a mental health biomedical research centre – a qualitative study. Res Involv Engagem.

[20] Hamilton C.B., Hoens A.M., Backman C.L. (2018). An empirically based conceptual framework for fostering meaningful patient engagement in research. Health Expect.

[21] Baines R., Bradwell H., Edwards K. (2022). Meaningful patient and public involvement in digital health innovation, implementation and evaluation: a systematic review. Health Expect.

[22] Miller F.A., Patton S.J., Dobrow M., Berta W. (2018). Public involvement in health research systems: a governance framework. Health Res Policy Syst.

[23] Caron-Flinterman J.F., Broerse J.E.W., Bunders J.F.G. (2007). Patient partnership in decision-making on biomedical research: changing the network. Sci Technol Hum Values.

[24] Ferrari A., Stark D., Peccatori F.A. (2021). Adolescents and young adults (AYA) with cancer: a position paper from the AYA Working Group of the European Society for Medical Oncology (ESMO) and the European Society for Paediatric Oncology (SIOPE). ESMO Open.

[25] Trama A., Stark D., Bozovic-Spasojevic I. (2023). Cancer burden in adolescents and young adults in Europe. ESMO Open.

[26] Sankaran H., Finnigan S.R., McShane L.M., Best A.F., Seibel N.L. (2022). Enrollment of adolescent and young adult patients newly diagnosed with cancer in NCI CTEP-sponsored clinical trials before and after launch of the NCI National Clinical Trials Network. Cancer.

[27] Husson O., Reeve B.B., Darlington A.S. (2022). Next step for global adolescent and young adult oncology: a core patient-centered outcome set. J Natl Cancer Inst.

[28] Košir U. (2020). Methodological issues in psychosocial research in adolescent and young adult cancer populations. J Adolesc Young Adult Oncol.

[29] Burgers V.W.G., Dickhout A., Harthoorn N.C.G.L. (2023). What makes patient involvement work? Lessons learned from a qualitative study in adolescents and young adults with cancer. Patient Educ Couns.

[30] Cheung C.K., Tucker-Seeley R., Davies S. (2021). A call to action: antiracist patient engagement in adolescent and young adult oncology research and advocacy. Future Oncol.

